# Comprehensive Analysis of DNA 5-Methylcytosine and N6-Adenine Methylation by Nanopore Sequencing in Hepatocellular Carcinoma

**DOI:** 10.3389/fcell.2022.827391

**Published:** 2022-03-07

**Authors:** Lili Zhang, Weiqi Rong, Jie Ma, Hexin Li, Xiaokun Tang, Siyuan Xu, Luyao Wang, Li Wan, Qing Zhu, Boyue Jiang, Fei Su, Hongyuan Cui

**Affiliations:** ^1^ Clinical Biobank, Beijing Hospital, National Center of Gerontology, Institute of Geriatric Medicine, Chinese Academy of Medical Sciences, Beijing, China; ^2^ Department of General Surgery, Department of Hepato-Bilio-Pancreatic Surgery, Beijing Hospital, National Center of Gerontology, Institute of Geriatric Medicine, Chinese Academy of Medical Sciences, Beijing, China; ^3^ Department of Hepatobiliary Surgery, National Cancer Center, National Clinical Research Center for Cancer, Cancer Hospital, Chinese Academy of Medical Sciences and Peking Union Medical College, Beijing, China; ^4^ Department of Hepatopancreatobiliary Surgery, Affiliated Hospital of Qinghai University, Qinghai, China

**Keywords:** 5-methylcytosine, N6-adenine methylation, nanopore sequencing, hepatocellular carcinoma, unstable methylation genes

## Abstract

DNA methylation is a widespread epigenetic signal in human genome. With Nanopore technology, differential methylation modifications including 5-methylcytosine (5mC) and 6-methyladenine (6mA) can be identified. 5mC is the most important modification in mammals, although 6mA may also function in growth and development as well as in pathogenesis. While the role of 5mC at CpG islands in promoter regions associated with transcriptional regulation has been well studied, but the relationship between 6mA and transcription is still unclear. Thus, we collected two pairs of tumor tissues and adjacent normal tissues from hepatocellular carcinoma (HCC) surgical samples for Nanopore sequencing and transcriptome sequencing. It was found that 2,373 genes had both 5mC and 6mA, along with up- and down-regulated methylation sites. These genes were regarded as unstable methylation genes. Compared with 6mA, 5mC had more inclined distribution of unstable methylation sites. Chi-square test showed that the levels of 5mC were consistent with both up- and down-regulated genes, but 6mA was not significant. Moreover, the top three unstable methylation genes, TBC1D3H, CSMD1, and ROBO2, were all related to cancer. Transcriptome and survival analyses revealed four potential tumor suppressor genes including KCNIP4, CACNA1C, PACRG, and ST6GALNAC3. In this study, we firstly proposed to combine 5mC and 6mA methylation sites to explore functional genes, and further research found top of these unstable methylation genes might be functional and some of them could serve as potential tumor suppressor genes. Our study provided a new solution for epigenetic regulation research and therapy of HCC.

## 1 Introduction

Hepatocellular carcinoma (HCC) is a common malignant tumor and also the fourth leading cause of cancer-related death worldwide (Kole et al., 2020; Sung et al., 2021). Although the exact etiologies of HCC remain unclear, both acute and chronic infections with hepatitis B virus (HBV) and hepatitis C virus (HCV) can be the major causes. Hepatitis can lead to liver cirrhosis and HCC (Ringelhan et al., 2017). Some HCC patients may benefit from current treatment strategies including radiofrequency ablation, surgery, liver transplantation, and immunotherapy (Kole et al., 2020). Traditionally, genetic instability is regarded as one of the most common events in HCC. Recent studies have revealed that HCC can be triggered by epigenetic modifications (Sceusi et al., 2011).

In mammal genomes, cytosine DNA methylation (5-methylcytosine, 5mC) is a widespread form of methylation and can function by directly regulating the occurrence and development of cancers (Mo et al., 2020; Lowe et al., 2021). In HCC, 5mC is closely associated with survival rates and prognosis (Hlady et al., 2019; Mo et al., 2020). Recent evidence has accumulated that the regulation of 5mC-mediated gene silencing usually operate via CpG islands methylation, while most CpG islands associated with gene promoters are rarely methylated (de Mendoza et al., 2019). It is inferred that gene body 5mC also positively influences gene expression, which is targeted by DNMT3 in PWWP domain, and the PWWP domain also attracts an active transcription factor H3K36me3 (Baubec et al., 2015; Morselli et al., 2015). Thus, gene body 5mC may prime for active transcription but are not predictive. The 5mC derived from cell-free DNA can be markers in HCC (Hlady et al., 2019), reveals potential advantages of methylation to the development of liquid biopsy.

N6-adenine methylation (6mA), initially a marker of DNA modification in prokaryotes, has been identified in eukaryotes, especially in mammalian and plant genomes. 6mA plays a role in growth, development, and tumor progression (Zhou et al., 2018). The whole 6mA modification map in human has been obtained via SMRT sequencing, confirming that 6mA is widespread in nuclear genome and mitochondrial genome (Xiao et al., 2018). Further studies have shown the amount of 6mA is extremely low in eukaryote, while the modification of 6mA in mammals is mainly concentrated on the mitochondrial DNA (Kawarada et al., 2017). However, the small number of sites does not mean they are not functional. For example, in c-kit gene, a single 5mC methylation site is enough to affect the binding of the transcription factor GATA-1 to the gene body and regulate the normal development of hematopoietic system (Yang et al., 2020). During the development of mouse trophoblast stem cells, the 6mA-mediated repression of stress-induced DNA double helix destabilization-SATB1 interactions is essential for gene regulation. 6mA can balance the boundaries between euchromatin and heterochromatin (Li et al., 2020), and knockdown of 6mA methyltransferase also affects transcriptome-wide fluctuation of gene expression (Luo et al., 2018). Thus, there are connections between DNA modification and gene expression, although the specific regulatory mechanisms are still uncertain.

Differential methylation can be identified by using long-sequencing nanopore technology. We selected methylation sites that are different between tumor and adjacent normal tissues. Chi-square test revealed that the levels of 5mC were significantly correlated with gene transcription, but 6mA didn’t show much relevance. In HCC, 2,373 genes had both 5mC and 6mA, with up- and down-regulated methylation sites. We considered these genes as unstable methylation genes. Since 5mC and 6mA can influence gene expressions, unstable methylation genes with top amount of methylation sites were selected. Based on transcriptome information, we found eleven genes in top 100 unstable methylation genes could affect the prognosis of patients and four of them could be potential tumor suppressor genes.

## 2 Materials and Methods

### 2.1 Patients and Samples

As sequencing samples, two pairs of tumor tissues and adjacent normal tissues were collected from HCC surgical samples at the Cancer Hospital of Chinese Academy of Medical Sciences. The specimens were immediately stored at −80°C. The tumor tissues comprised >80% malignant cells, and the normal tissues comprised a mixture of normal epithelial cells and stromal cells. The constructed Nanopore sequencing library and RNA library were same as in our previous research (Zhuo et al., 2021). All the patients signed informed consent forms, and the research was approved by the Ethics Committee of Beijing Hospital.

### 2.2 Nanopore Sequencing and Illumina Sequencing

For Nanopore sequencing, DNA in tissues was extracted by using MagAttract HMW DNA kit (Qiagen). The double-stranded DNA was quantified by Nanodrop 2000 and Qubit dsDNA HS analysis kits (Thermo Fisher Scientific). AMPure XP (Beckman Coulter) and Qubit®3.0 fluorometer (Life Technologies) were used to purify and concentrate DNA. The Nanopore sequencing platform was GridION, R9.4.1 chip (ONT). After the quality of chip was checked in accordance with the manufacturer’s instructions, samples were sequenced. MinKNOW (v3.5.10) and Guppy (v3.2.6) software were used to collect raw electronic signals and convert the files to FASTQ format.

For Illumina sequencing, the DynabeadsTM mRNA Purification Kit (Invitrogen) was used to extract mRNA from total RNA. Ribo-Zero Gold Kits were utilized to remove rRNA. According to the instructions of the NEB-Next Ultra Directional RNA Library Prep Kit for Illumina (NEB, Ispawich, USA), different index tags were selected. The constructed libraries were sequenced using Illumina with 150 bp paired-end reads.

### 2.3 Methylation Calling for Nanopore Reads

For 5mC, Minimap2 (Li, 2018) was used to align sequencing reads to human genome (GRCh37). Nanopolish call-methylation (Simpson et al., 2017) was used to determine the methylation status of CpG site. The “-s” option in Nanopolish script was used to split group into individual sites.

For 6mA, we aligned data by using the re-squiggle algorithm in Tombo (version 1.4) (https://nanoporetech.github.io/tombo/). The alternative DNA models were available in Tombo and the 6mA model was used. The method identified 6mA methylation sites better than the canonical expected levels, which signal matches a specific alternative base expected signal levels.

After the methylation signal scores of each genome sites were obtained, we removed sites with all zero scores. The scores ranged from 0 to 1, in which 0 means completely unmethylated and 1 means completely methylated. Only sites whose methylation score changed by greater than 2 folds were considered. When scores in part of tissues were zero, the sites with scores greater than 0.6 in the remaining tissues were defined as different sites. The sites with significantly different scores were regarded as unstable methylation sites. We used Bedtools intersect (Quinlan and Hall, 2010) to annotate genes corresponding to unstable methylation sites. The distribution of methylation sites in genome was drawn by package CMplot (Yin et al., 2021) in R language. The methylation intersection map used VennDetail-Shiny (http://hurlab.med.und.edu:3838/VennDetail/).

In order to verify the accuracy of methylation sites, Megalodon (https://github.com/nanoporetech/megalodon) was applied to reanalyze the sequencing data based on the previous studies (Yuen et al., 2021). The intersection of two data sets from different methods was obtained. The methylation model was downloaded from Rerio (https://github.com/nanoporetech/rerio) as recommended “res_dna_r941_min_modbases-all-context_v001”. In order to verify the accuracy of methylation analysis, the PCR amplified fragments of top unstable methylation genes and tumor suppressor genes were performed on Nanopore (Supplementary Figure S1), and the sequencing data were analyzed parallelly. The sequences generated by PCR amplification did not have any methylation sites (Supplementary Figure S2) which proved the reliability of our data processes.

### 2.4 Differential Gene Expression

FastQC (Andrews, 2010) was used to evaluated the quality of sequencing data, which revealed some reads mixed with adapters. In order to filter reads, we used Trimmomatic (Bolger et al., 2014). The software dropped reads less than 28 nt, and the average quality was less than 15 through a four-base sliding window. After the data was qualified, the reads were mapped to genome by STAR (Dobin et al., 2013). For each gene, we chose featureCounts (Liao et al., 2014) to quantify read counts. The parameter “requireBothEndsMapped” and “isPairedEnd” were set TRUE. By DGEList and rpkm function in package edgeR (Robinson et al., 2010), we calculated the normalized expression levels of genes. We chose genes with fold-change greater than 2 as differential expressed genes.

### 2.5 Functional Enrichment Analysis and Survival Analysis

The R package ClusterProfiler (Wu et al., 2021) was used to analyse gene set enrichment. It queried the latest online database to perform functional analysis and allowed the output up-to-date. The pathways were drawn by using ggplot2 (Wickham, 2016).

For survival analysis, we used TCGA liver cancer (LIHC) in UCSC Xena (Goldman et al., 2020) with overall survival to get Kaplan-Meier plots of unstable methylation genes. The “*p*-value < 0.05” genes in survival analysis were selected.

## 3 Result

### 3.1 The Distribution of Methylation Sites in Genome

**FIGURE 1:**
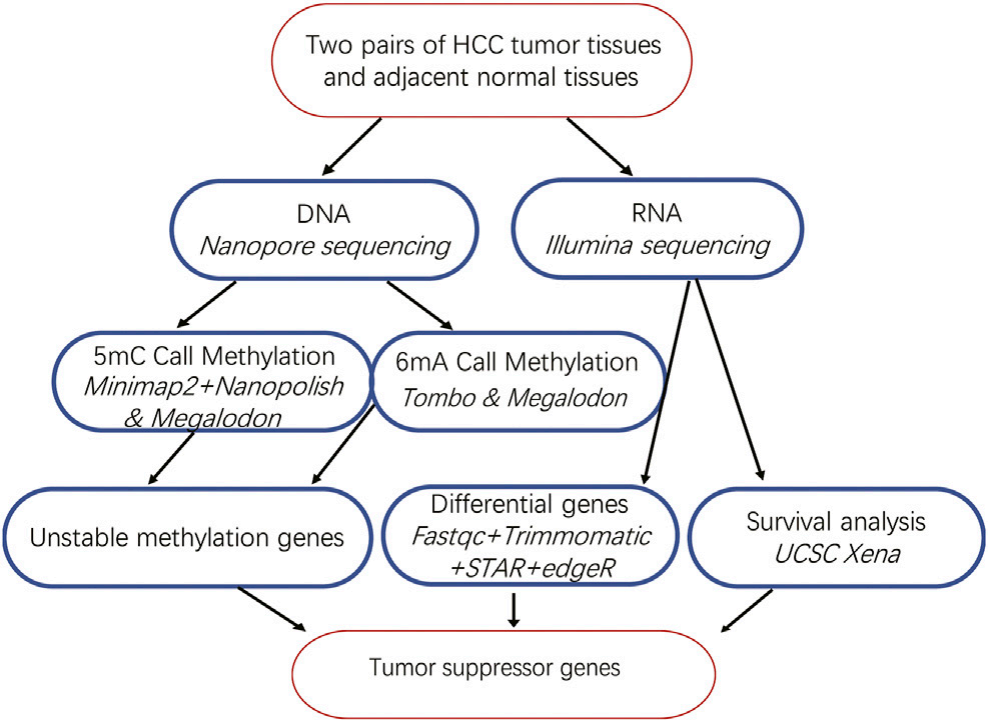
Bioinformatics analysis pipeline of our study.

**FIGURE 2:**
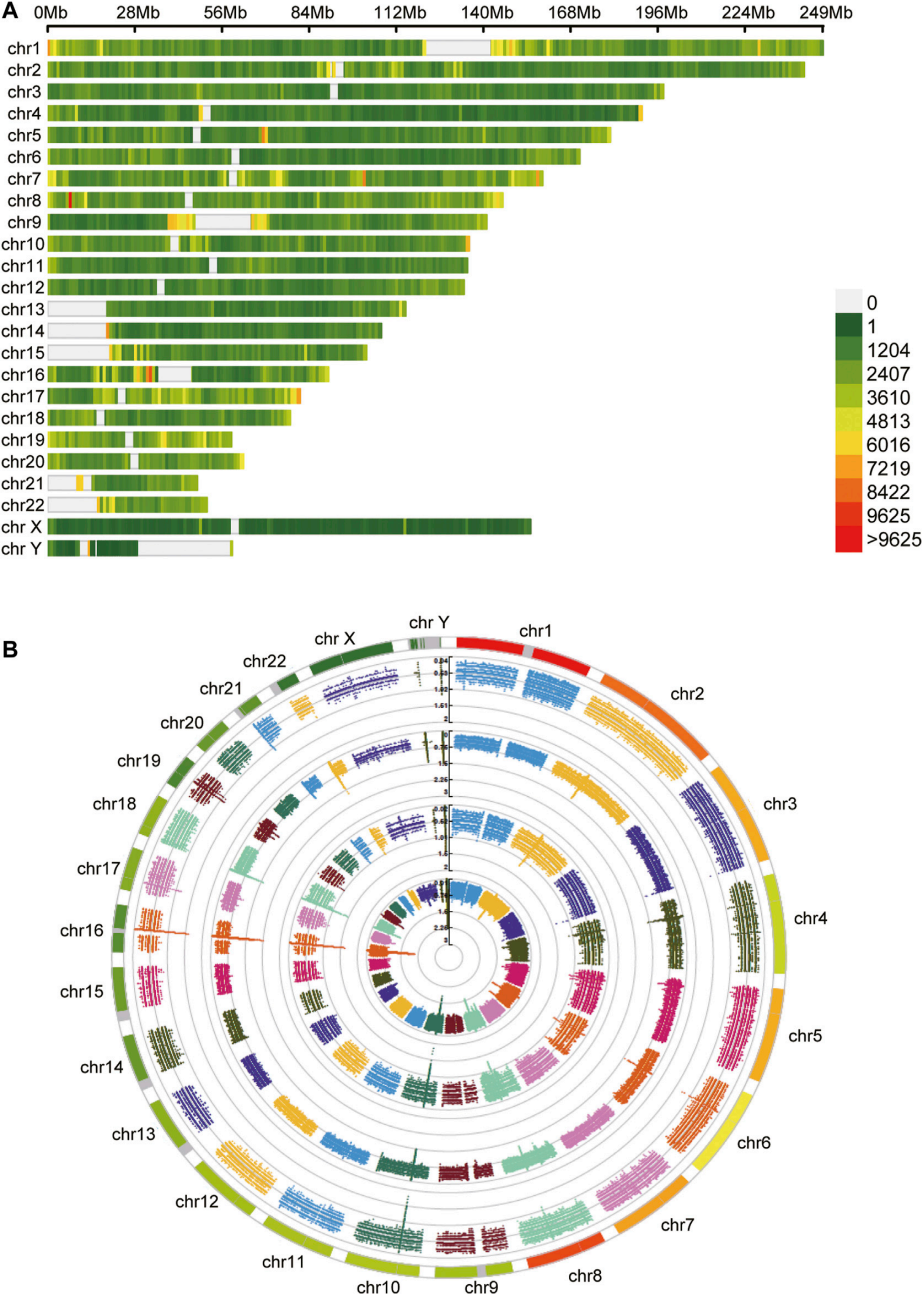
The distribution of methylation sites. **(A)** The 5mC unstable methylation sites in genome. The gap regions belong to genome centromere region. **(B)** The 6mA unstable methylation sites in genome.

**FIGURE 3:**
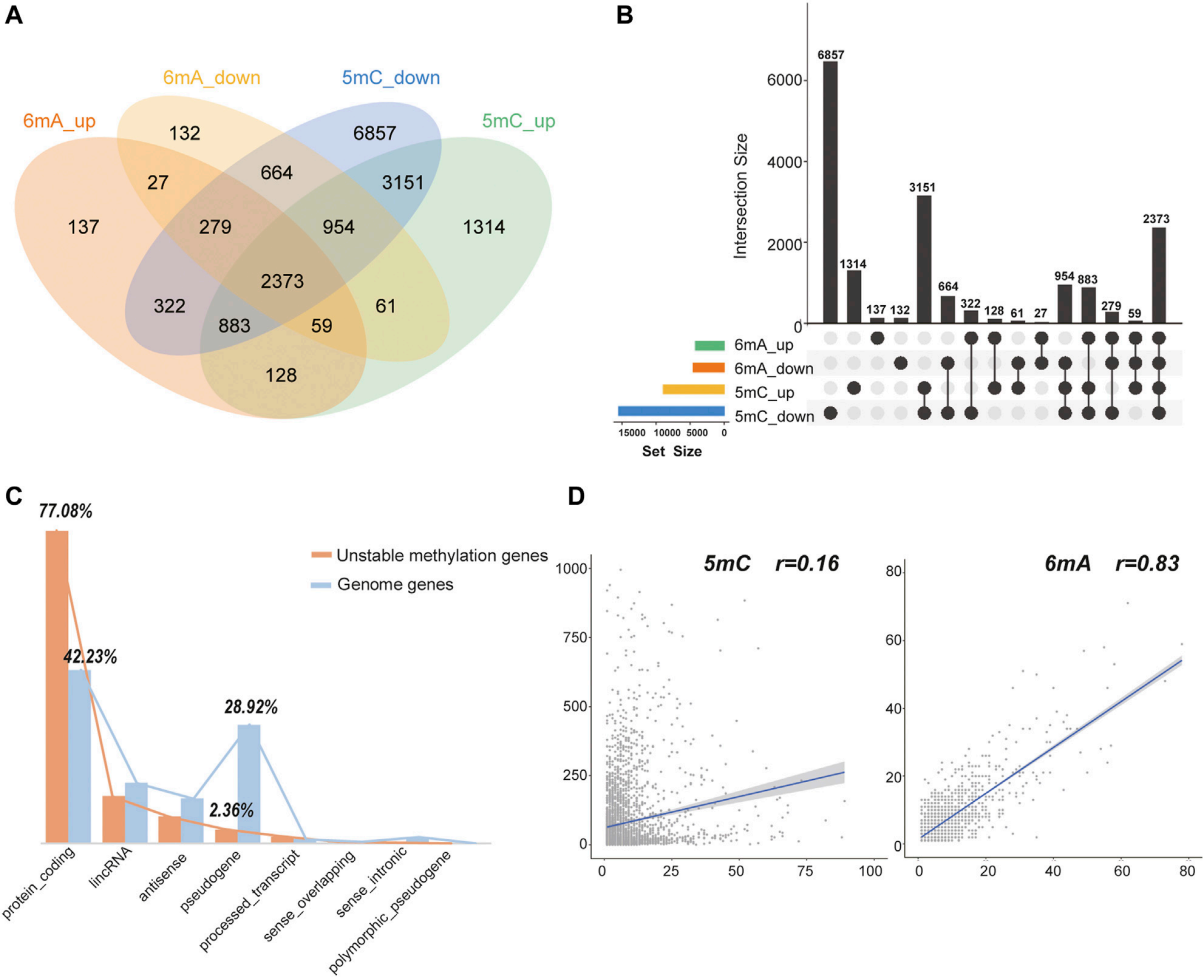
The statistics of 5mC and 6mA methylation changed sites. **(A,B)** The intersection number of genes with 5mC and 6mA, up- and down-regulated methylation sites. **(C)** The gene types of unstable methylation genes. The orange column represented unstable methylation genes, and the blue column represented genes in human genome. **(D)** The Pearson correlation coefficient between the number of up-regulated and down-regulated sites in 5mC and 6mA. It is only 0.16 for 5mC but 0.83 for 6mA.

After obtaining tumor and adjacent normal tissues, we constructed sequencing libraries and developed analysis pipeline (Figure 1). Output of sequencing data showed the wide distribution of 5mC and 6mA sites in the genome (Figure 2). Except for centromere region which full of repetitive sequences affect detection, both 5mC and 6mA were widely distributed in the genome. For the comparison of the methylation signals between HCC tumor and adjacent normal liver tissues, we extracted 5mC and 6mA sites that were significantly different and compared the methylation transition of these sites and their genes (Figure 3). Since the change of a single site may affect gene expression (Yang et al., 2020), we recalled all the sites with methylation score changed at the gene level. The filtered up-regulated or down-regulated sites were the methylation signals with scores changed by more than two folds. For genes that might be affected, the amount of 5mC changed was significantly higher than that of 6mA (Figures 3A,B). Notably, there were 2,373 genes having both own 5mC and 6mA, with up- and down-regulated methylation sites (Supplementary File S1). These genes were considered as unstable methylation genes in HCC.

Further analysis showed that 77.08% of these unstable methylation genes belonged to protein-coding genes, which was much higher than 42.23% of protein coding genes in genome. While 28.92% of pseudogenes were found in genome, the proportion of 5mC and 6mA changed was only 2.36% (Figure 3C). Thus, genes with unstable methylation were more likely to be protein-coding genes, and these unstable methylation genes might have regulatory functions in transcription and translation. In addition, the Pearson correlation coefficient between the number of up-regulated and down-regulated sites in 6mA was 0.83, while the correlation coefficient in 5mC was only 0.16 (Figure 3D). It demonstrates the distribution of 5mC was more specific than mA. In addition, 45.47% of these unstable methylation genes had altered transcriptome suggesting the methylation in 5mC site was more specific than 6mA.

### 3.2 Relationship Between Methylation and Transcription

**FIGURE 4:**
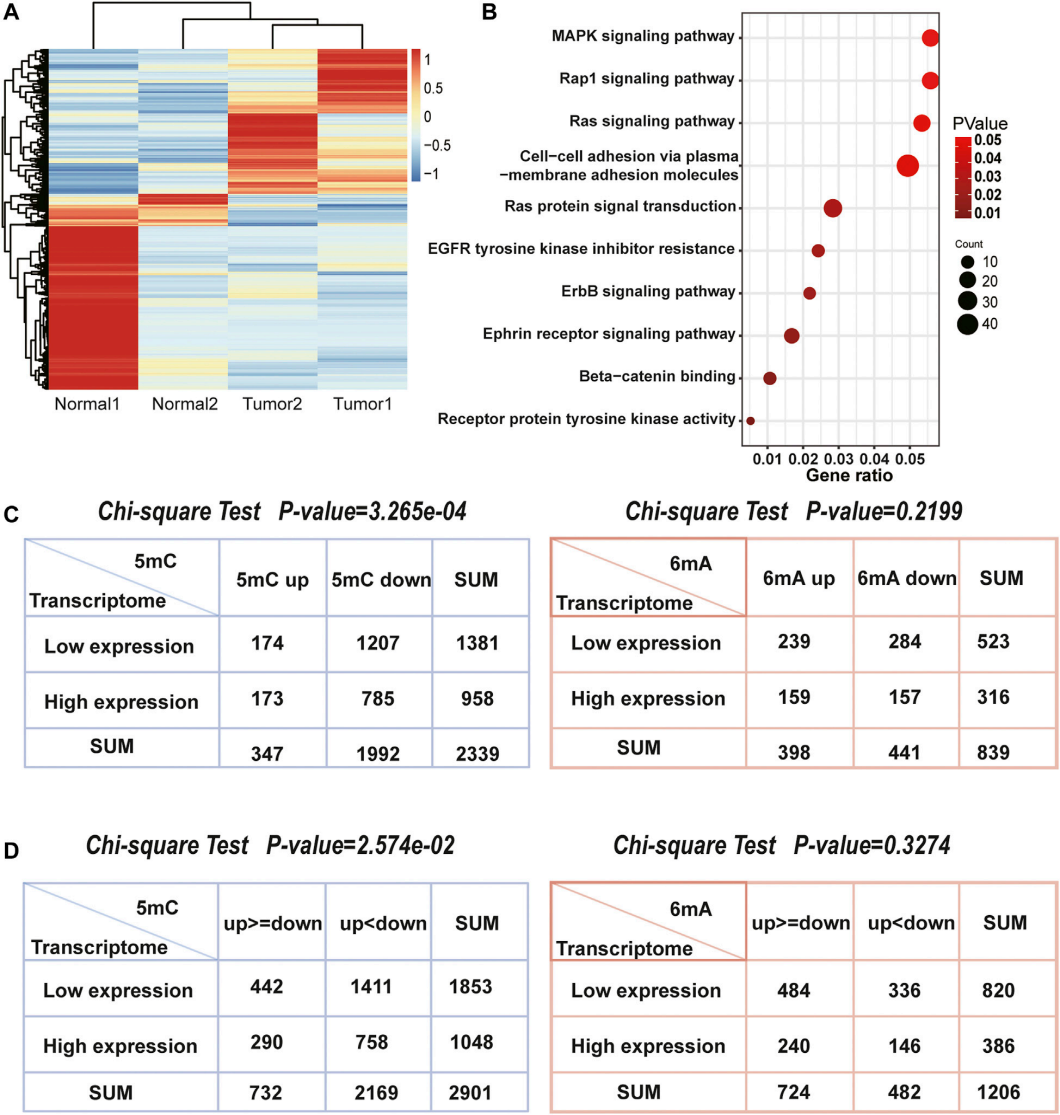
The association between differentially expressed genes and methylation related genes. **(A)** Heatmap of two pairs transcriptome sequencing data. **(B)** The enriched pathways of differentially expressed genes in HCC. They were closely associated with HCC. **(C)** The number of 5mC, 6mA genes and differentially expressed genes. The Chi-square test indicated that 5mC were significantly associated with differentially expressed genes, but 6mA were not. **(D)** The number of genes both own 5mC or 6mA up and down methylation sites, as well as differentially expressed genes. The Chi-square test also indicated that 5mC were more related with gene transcription than 6mA.

After acquiring the sequencing data, we focused on transcriptome (Figure 4A) and found the differentially expressed genes were indeed enriched in pathways related to liver cancer (Figure 4B). Furthermore, Chi-square test was used to explore whether 5mC, 6mA were related to differentially expressed genes. It showed that the amount of differentially expressed genes was highly correlated with 5mC-modifications instead of 6mA which is also consistent with the correlation of up- and down-regulated sites in Figure 3D. In HCC, 1,470 genes had specifically up-regulated 6mA sites. The amount of the specific down-regulated 6mA genes was 1,811. Totally, there were 1,417 differentially expressed genes. After classification, Chi-square test indicated that the specifically regulated 5mC genes instead of 6mA were significantly associated with differentially expressed genes (Figure 4C). Similarly, the significant difference of 5mC was also observed with the *p*-value of 3.265e-04 (Figure 4C). The results indicated that in HCC patients, 5mC methylation plays more important role than 6mA in differential transcription.

While genes possess both up and downregulated methylation signals, it is worth to note that whether more methylation signals have more influence on differentially expressed genes, such as genes with more upregulated methylation sites tend to be low-expressed in transcriptome. Thus, we counted 2,901 genes with 5mC up and downregulated methylation signals and 1,206 genes with 6mA (Figure 4D). Chi-square test also demonstrated that the levels of 5mC were consistent with both up and downregulated genes, but 6mA was not significant. Although the relationship between the level of 5mC and the mRNA was not linear, the influence of 5mC on transcription is more remarkable than 6mA. It revealed that DNA methylation on the 5mC plays a more important role than 6mA in transcriptional regulation.

### 3.3 Unstable Methylation Genes and Their Relationships With Survival

DNA methylation accumulation and the epigenotype formation on the genome may occur in the early stages of carcinogenesis and can predict the future cancer type (Kaneda et al., 2014). Therefore, genes with more accumulated methylation changed sites may play roles in the occurence and development of cancer. In order to explore HCC-related unstable methylation genes, we combined HCC transcriptome data and TCGA survival analysis.

In HCC, the genes at top list of unstable methylation sites are TBC1D3H, CSMD1 and ROBO2. TBC1D3H belongs to TBC1 domain family, which can act as a GTPase activating protein for RAB5 (Itoh et al., 2006; UniProt, 2021). Its dysregulation can lead to tumorigenesis (Jian et al., 2020). Other proteins with TBC1 domain also function in cancer; for instance, TBC1 domain family member 23 can interact with Ras-related protein Rab-11A to promote poor prognosis in lung cancer (Zhang et al., 2021). The second unstable methylation gene is CSMD1. Deregulation of CSMD1 can link inflammation to carcinogenesis via activating NF-κB pathway. The process subsequently leads to the upregulation of c-Myc and epithelial-mesenchymal transition markers (Chen et al., 2019). It has also been reported that combined identification of ARID1A, SENP3, and CSMD1 are effective prognostic biomarkers for HCC patients (Zhao et al., 2021). The third gene RoBo2 can suppress cancer development through TGF-β signalling and stroma activation (Pinho et al., 2018). The evidence showed that the top unstable methylation genes are involved in the occurrence and development of cancer.

**FIGURE 5:**
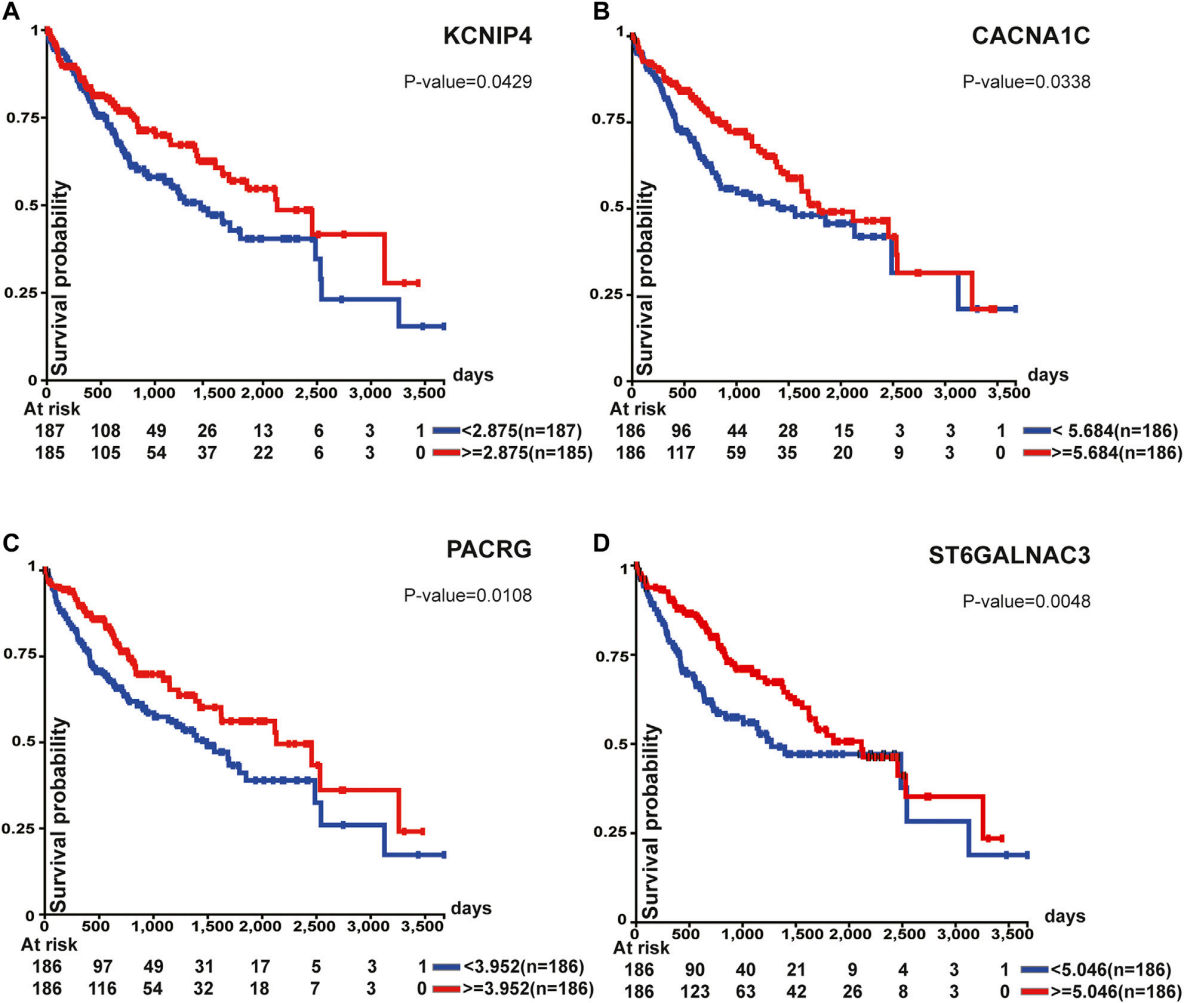
Survival analysis of tumor suppressor genes. All of these genes were associated with overall survival.

Survival analysis of top 100 unstable methylation genes was performed. As shown by the Kaplan-Meier plot (*p*-value<0.05), 11 genes were significantly associated with overall survival of patients (Supplementary File S1). Downregulation of tumor suppressor genes function importantly in cancer formation and progression, which involves alteration of epigenetic modifications in genome-wide (Davenport et al., 2021). Combined with transcriptome, six genes could be regarded as tumor suppressor genes (Figure 5). Among them, CTNNA3 was reported as a tumor suppressor in HCC before (He et al., 2016) and the same as DLG2 in osteosarcoma (Shao et al., 2019). The other four identified genes were KCNIP4, CACNA1C, PACRG, and ST6GALNAC3 (Figure 5). For KCNIP4, its related pathways were regulation of Wnt-mediated β-catenin signaling and target gene transcription (Kitagawa et al., 2007), in which the elevated Wnt-mediated β-catenin signaling could enhance the proliferation of liver cells in HCC (Wang et al., 2019). CACNA1C could be a prognostic predictor in ovarian cancer (Chang and Dong, 2021), and its overall survival was equally significant in HCC (Figure 5B). Abnormal promoter methylation of PACRG (Agirre et al., 2006), and ST6GALNAC3 (Haldrup et al., 2018) were associated with downregulation of gene expression in cancers. However, methylation was not limited to gene promoter. We found the unstable methylation sites in their gene body also changed markedly. Therefore, some of the unstable methylation genes may also serve as tumor suppressor genes, providing new ideas for mining tumor-related genes in future.

## 4 Discussion

Compared with the well elucidated mechanism of 5mC on transcriptional regulation, other DNA methylation modification remains uncertain. It has been reported that 6mA is complementary to 5mC as an epigenomic mark in rice (Zhou et al., 2018). Thus, we analyzed Nanopore sequencing data to observe whether there is a clearly transcriptional regulatory mechanism for 5mC and 6mA. Our study found that 6mA had less influence on gene expression than 5mC. The Pearson correlation coefficient of the number of up and downregulated sites of 5mC was 0.16, and that of 6mA was 0.83. The distribution of 5mC up and downregulated sites was more inclined and the distribution of 6mA was more “uniform”. There were 2,373 unstable methylation genes having both 5mC and 6mA, with up and downregulated methylation sites. The expressions of 1371 genes were different in tumor tissues and adjacent normal tissues. The statistics for 1371 genes did not prove obviously complementarity. It might be due to the species different. The number of genes with 5mC showed significant correlation with the number of differentially expressed genes, although such correlation was not linear. Meanwhile, 6mA had less effect on transcription, which requires further studies.

These 2,373 genes were regarded as unstable methylation genes, and their expressions were related to the number of up and downregulated methylation sites within them. In order to explore HCC related genes, we combined the transcriptome and survival data of TCGA liver cancer. Among the top 100 unstable methylation genes, we found eleven genes significantly affected the prognosis, and four of them can be defined as tumor suppressor genes. Normally, tumor suppressor genes can inhibit tumor cell proliferation and development. A typical tumor suppressor gene often occurs genetic alterations or epigenetic abnormality that reduce gene expression (Guo et al., 2010). While they are expressed at low level or inactivated in tumors, cell growth may lose control and facilitate tumor progression. Decreased expression of tumor suppressor gene also correlates with poor prognosis and reduced survival. Thus, the newly found tumor suppressor genes in this study can be prognostic predictor in HCC.

The top three unstable methylation genes in HCC are TBC1D3H, CSMD1, and ROBO2, which are closely related to the occurrence and development of HCC. TBC1D3H possesses TBC1 domain, and the proteins of this family were reported to regulate GTPase activation, which is related to tumorigenesis. CSMD1 is involved in such well-known tumor pathways as NF-κB pathway and epithelial-mesenchymal transition pathway. ROBO2 can suppress cancer development. Thus, genes with more unstable methylation sites in HCC are closely related to tumors. For the four newly discovered tumor suppressor genes, i.e., KCNIP4, CACNA1C, PACRG, and ST6GALNAC3, previous studies have proved their regulatory effect in tumors. These four genes were firstly considered to be tumor suppressor genes through methylation site screening. DNA methylation plays a critical interaction between tumor and immune cells. Tumor cells can escape immune restriction by various epigenetic mechanisms including DNA methylation (Cao and Yan, 2020). The top unstable methylation genes in HCC could be the pharmaceutical candidates like epigenetic regulators. The repair of aberrant methylation may trigger antitumor immune responses and further improve immunological surveillance. The targeting agents of unstable methylation genes will have major impacts in tumor-related treatment.

## Data Availability

The datasets presented in this study can be found in online repositories. The names of the repository/repositories and accession number(s) can be found below: https://ngdc.cncb.ac.cn/gsa-human/browse/HRA001037, HRA001037.

## References

[B1] Agirre X. Román-Gómez J. Vázquez I. Jiménez-Velasco A. Garate L. Montiel-Duarte C. (2006). Abnormal Methylation of the commonPARK2andPACRGpromoter Is Associated with Downregulation of Gene Expression in Acute Lymphoblastic Leukemia and Chronic Myeloid Leukemia. Int. J. Cancer 118 (8), 1945–1953. 10.1002/ijc.21584 16287063

[B48] Andrews S. (2010). Fastqc: A Quality Control Tool for High Throughput Sequence Data. Available at: https://www.bioinformatics.babraham.ac.uk/projects/fastqc/ (Accessed January 10, 2022).

[B2] Baubec T. Colombo D. F. Wirbelauer C. Schmidt J. Burger L. Krebs A. R. (2015). Genomic Profiling of DNA Methyltransferases Reveals a Role for DNMT3B in Genic Methylation. Nature 520 (7546), 243–247. 10.1038/nature14176 25607372

[B3] Bolger A. M. Lohse M. Usadel B. (2014). Trimmomatic: a Flexible Trimmer for Illumina Sequence Data. Bioinformatics 30 (15), 2114–2120. 10.1093/bioinformatics/btu170 24695404 PMC4103590

[B4] Cao J. Yan Q. (2020). Cancer Epigenetics, Tumor Immunity, and Immunotherapy. Trends Cancer 6 (7), 580–592. 10.1016/j.trecan.2020.02.003 32610068 PMC7330177

[B5] Chang X. Dong Y. (2021). CACNA1C Is a Prognostic Predictor for Patients with Ovarian Cancer. J. Ovarian Res. 14 (1), 88. 10.1186/s13048-021-00830-z 34210324 PMC8252246

[B6] Chen X.-L. Hong L.-L. Wang K.-L. Liu X. Wang J.-L. Lei L. (2019). Deregulation of CSMD1 Targeted by microRNA-10b Drives Gastric Cancer Progression through the NF-Κb Pathway. Int. J. Biol. Sci. 15 (10), 2075–2086. 10.7150/ijbs.23802 31592231 PMC6775299

[B7] Davenport C. F. Scheithauer T. Dunst A. Bahr F. S. Dorda M. Wiehlmann L. (2021). Genome-Wide Methylation Mapping Using Nanopore Sequencing Technology Identifies Novel Tumor Suppressor Genes in Hepatocellular Carcinoma. Ijms 22 (8), 3937. 10.3390/ijms22083937 33920410 PMC8069345

[B8] de Mendoza A. Lister R. Bogdanovic O. (2020). Evolution of DNA Methylome Diversity in Eukaryotes. J. Mol. Biol. 432, 1687–1705. 10.1016/j.jmb.2019.11.003 31726061

[B9] Dobin A. Davis C. A. Schlesinger F. Drenkow J. Zaleski C. Jha S. (2013). STAR: Ultrafast Universal RNA-Seq Aligner. Bioinformatics 29 (1), 15–21. 10.1093/bioinformatics/bts635 23104886 PMC3530905

[B10] Goldman M. J. Craft B. Hastie M. Repečka K. McDade F. Kamath A. (2020). Visualizing and Interpreting Cancer Genomics Data via the Xena Platform. Nat. Biotechnol. 38 (6), 675–678. 10.1038/s41587-020-0546-8 32444850 PMC7386072

[B11] Guo X. Liu W. Pan Y. Ni P. Ji J. Guo L. (2010). Homeobox Gene IRX1 Is a Tumor Suppressor Gene in Gastric Carcinoma. Oncogene 29 (27), 3908–3920. 10.1038/onc.2010.143 20440264

[B12] Haldrup C. Pedersen A. L. Øgaard N. Strand S. H. Høyer S. Borre M. (2018). Biomarker Potential ofST6GALNAC3andZNF660promoter Hypermethylation in Prostate Cancer Tissue and Liquid Biopsies. Mol. Oncol. 12 (4), 545–560. 10.1002/1878-0261.12183 29465788 PMC5891052

[B13] He B. Li T. Guan L. Liu F.-E. Chen X.-M. Zhao J. (2016). CTNNA3 Is a Tumor Suppressor in Hepatocellular Carcinomas and Is Inhibited by miR-425. Oncotarget 7 (7), 8078–8089. 10.18632/oncotarget.6978 26882563 PMC4884977

[B14] Hlady R. A. Zhao X. Pan X. Yang J. D. Ahmed F. Antwi S. O. (2019). Genome-wide Discovery and Validation of Diagnostic DNA Methylation-Based Biomarkers for Hepatocellular Cancer Detection in Circulating Cell Free DNA. Theranostics 9 (24), 7239–7250. 10.7150/thno.35573 31695765 PMC6831291

[B15] Itoh T. Satoh M. Kanno E. Fukuda M. (2006). Screening for Target Rabs of TBC (Tre-2/Bub2/Cdc16) Domain-Containing Proteins Based on Their Rab-Binding Activity. Genes Cells 11 (9), 1023–1037. 10.1111/j.1365-2443.2006.00997.x 16923123

[B16] Jian Z. Zhang L. Jin L. Lan W. Zhang W. Gao G. (2020). Rab5 Regulates the Proliferation, Migration and Invasion of Glioma Cells via Cyclin E. Oncol. Lett. 20 (2), 1055–1062. 10.3892/ol.2020.11660 32724343 PMC7377158

[B17] Kaneda A. Matsusaka K. Sakai E. Funata S. (2014). DNA Methylation Accumulation and its Predetermination of Future Cancer Phenotypes. J. Biochem. 156 (2), 63–72. 10.1093/jb/mvu038 24962701

[B18] Kawarada L. Suzuki T. Ohira T. Hirata S. Miyauchi K. Suzuki T. (2017). ALKBH1 Is an RNA Dioxygenase Responsible for Cytoplasmic and Mitochondrial tRNA Modifications. Nucleic Acids Res. 45 (12), 7401–7415. 10.1093/nar/gkx354 28472312 PMC5499545

[B19] Kitagawa H. Ray W. J. Glantschnig H. Nantermet P. V. Yu Y. Leu C.-T. (2007). A Regulatory Circuit Mediating Convergence between Nurr1 Transcriptional Regulation and Wnt Signaling. Mol. Cell Biol 27 (21), 7486–7496. 10.1128/MCB.00409-07 17709391 PMC2169041

[B20] Kole C. Charalampakis N. Tsakatikas S. Vailas M. Moris D. Gkotsis E. (2020). Immunotherapy for Hepatocellular Carcinoma: A 2021 Update. Cancers 12 (10), 2859. 10.3390/cancers12102859 PMC7600093 33020428

[B21] Li H. (2018). Minimap2: Pairwise Alignment for Nucleotide Sequences. Bioinformatics 34 (18), 3094–3100. 10.1093/bioinformatics/bty191 29750242 PMC6137996

[B22] Li Z. Zhao S. Nelakanti R. V. Lin K. Wu T. P. Alderman M. H. 3rd (2020). N6-methyladenine in DNA Antagonizes SATB1 in Early Development. Nature 583 (7817), 625–630. 10.1038/s41586-020-2500-9 32669713 PMC8596487

[B23] Liao Y. Smyth G. K. Shi W. (2014). featureCounts: an Efficient General Purpose Program for Assigning Sequence Reads to Genomic Features. Bioinformatics 30 (7), 923–930. 10.1093/bioinformatics/btt656 24227677

[B24] Lowe P. Olinski R. Ruzov A. (2021). Evidence for Noncytosine Epigenetic DNA Modifications in Multicellular Eukaryotes: An Overview. Methods Mol. Biol. 2198, 15–25. 10.1007/978-1-0716-0876-0_2 32822019

[B25] Luo G.-Z. Hao Z. Luo L. Shen M. Sparvoli D. Zheng Y. (2018). N6-methyldeoxyadenosine Directs Nucleosome Positioning in Tetrahymena DNA. Genome Biol. 19 (1), 200. 10.1186/s13059-018-1573-3 30454035 PMC6245762

[B26] Mo Z. Cao Z. Luo S. Chen Y. Zhang S. (2020). Novel Molecular Subtypes Associated with 5mC Methylation and Their Role in Hepatocellular Carcinoma Immunotherapy. Front. Mol. Biosci. 7, 562441. 10.3389/fmolb.2020.562441 33195409 PMC7645064

[B27] Morselli M. Pastor W. A. Montanini B. Nee K. Ferrari R. Fu K. (2015). *In Vivo* targeting of De Novo DNA Methylation by Histone Modifications in Yeast and Mouse. Elife 4, e06205. 10.7554/eLife.06205 25848745 PMC4412109

[B28] Pinho A. V. Van Bulck M. Chantrill L. Arshi M. Sklyarova T. Herrmann D. (2018). ROBO2 Is a Stroma Suppressor Gene in the Pancreas and Acts via TGF-β Signalling. Nat. Commun. 9 (1), 5083. 10.1038/s41467-018-07497-z 30504844 PMC6269509

[B29] Quinlan A. R. Hall I. M. (2010). BEDTools: a Flexible Suite of Utilities for Comparing Genomic Features. Bioinformatics 26 (6), 841–842. 10.1093/bioinformatics/btq033 20110278 PMC2832824

[B30] Ringehan M. McKeating J. A. Protzer U. (2017). Viral Hepatitis and Liver Cancer. Phil. Trans. R. Soc. B 372 (1732), 20160274. 10.1098/rstb.2016.0274 28893941 PMC5597741

[B31] Robinson M. D. McCarthy D. J. Smyth G. K. (2010). edgeR: a Bioconductor Package for Differential Expression Analysis of Digital Gene Expression Data. Bioinformatics 26 (1), 139–140. 10.1093/bioinformatics/btp616 19910308 PMC2796818

[B32] Sceusi E. L. Loose D. S. Wray C. J. (2011). Clinical Implications of DNA Methylation in Hepatocellular Carcinoma. Hpb 13 (6), 369–376. 10.1111/j.1477-2574.2011.00303.x 21609368 PMC3103092

[B33] Shao Y. W. Wood G. A. Lu J. Tang Q.-L. Liu J. Molyneux S. (2019). Cross-species Genomics Identifies DLG2 as a Tumor Suppressor in Osteosarcoma. Oncogene 38 (2), 291–298. 10.1038/s41388-018-0444-4 30093633 PMC6756098

[B34] Simpson J. T. Workman R. E. Zuzarte P. C. David M. Dursi L. J. Timp W. (2017). Detecting DNA Cytosine Methylation Using Nanopore Sequencing. Nat. Methods 14 (4), 407–410. 10.1038/nmeth.4184 28218898

[B35] Sung H. Ferlay J. Siegel R. L. Laversanne M. Soerjomataram I. Jemal A. (2021). Global Cancer Statistics 2020: GLOBOCAN Estimates of Incidence and Mortality Worldwide for 36 Cancers in 185 Countries. CA A. Cancer J. Clin. 71 (3), 209–249. 10.3322/caac.21660 33538338

[B36] UniProt C. (2021). UniProt: the Universal Protein Knowledgebase in 2021. Nucleic Acids Res. 49 (D1), D480–D489. 10.1093/nar/gkaa1100 33237286 PMC7778908

[B37] Wang W. Smits R. Hao H. He C. (2019). Wnt/β-Catenin Signaling in Liver Cancers. Cancers 11 (7), 926. 10.3390/cancers11070926 PMC6679127 31269694

[B38] Wickham H. (2016). ggplot2: Elegant Graphics for Data Analysis. New York: Springer-Verlag.

[B39] Wu T. Hu E. Xu S. Chen M. Guo P. Dai Z. (2021). clusterProfiler 4.0: A Universal Enrichment Tool for Interpreting Omics Data. The Innovation 2 (3), 100141. 10.1016/j.xinn.2021.100141 34557778 PMC8454663

[B40] Xiao C.-L. Zhu S. He M. ChenZhang D. Zhang Q. Chen Y. (2018). N6-Methyladenine DNA Modification in the Human Genome. Mol. Cell 71 (2), 306–318. 10.1016/j.molcel.2018.06.015 30017583

[B41] Yang L. Chen Z. Stout E. S. Delerue F. Ittner L. M. Wilkins M. R. (2020). Methylation of a CGATA Element Inhibits Binding and Regulation by GATA-1. Nat. Commun. 11 (1), 2560. 10.1038/s41467-020-16388-1 32444652 PMC7244756

[B42] Yin L. Zhang H. Tang Z. Xu J. Yin D. Zhang Z. (2021). rMVP: A Memory-Efficient, Visualization-Enhanced, and Parallel-Accelerated Tool for Genome-wide Association Study. Genomics, Proteomics & Bioinformatics. 10.1016/j.gpb.2020.10.007 PMC9040015 33662620

[B43] Yuen Z. W. Srivastava A. Daniel R. McNevin D. Jack C. Eyras E. (2021). Systematic Benchmarking of Tools for CpG Methylation Detection from Nanopore Sequencing. Nat. Commun. 12 (1), 3438. 10.1038/s41467-021-23778-6 34103501 PMC8187371

[B44] Zhang Y. Su H. Wudu M. Ren H. Xu Y. Zhang Q. (2021). TBC1 Domain Family Member 23 Interacts with Ras‐related Protein Rab‐11A to Promote Poor Prognosis of Non‐small‐cell Lung Cancer via β1‐integrin. J. Cell Mol Med 25 (18), 8821–8835. 10.1111/jcmm.16841 34363324 PMC8435452

[B45] Zhao Y. Yang B. Chen D. Zhou X. Wang M. Jiang J. (2021). Combined Identification of ARID1A, CSMD1, and SENP3 as Effective Prognostic Biomarkers for Hepatocellular Carcinoma. Aging 13 (3), 4696–4712. 10.18632/aging.202586 33558447 PMC7906131

[B46] Zhou C. Wang C. Liu H. Zhou Q. Liu Q. Guo Y. (2018). Identification and Analysis of Adenine N6-Methylation Sites in the rice Genome. Nat. Plants 4 (8), 554–563. 10.1038/s41477-018-0214-x 30061746

[B47] Zhuo Z. Rong W. Li H. Li Y. Luo X. Liu Y. (2021). Long-read Sequencing Reveals the Structural Complexity of Genomic Integration of HBV DNA in Hepatocellular Carcinoma. Npj Genom. Med. 6 (1), 84. 10.1038/s41525-021-00245-1 34642322 PMC8511263

