# Supplementary material for: Comprehensive Analysis of DNA 5-Methylcytosine and N6-Adenine Methylation by Nanopore Sequencing in Hepatocellular Carcinoma
Source: Front Cell Dev Biol. 2022 Mar 7;10:827391. doi: 10.3389/fcell.2022.827391 (PMC8937020; doi:10.3389/fcell.2022.827391)
Supplement: Supplementary file 1 [file DataSheet1.PDF]

The primer sequence：

ST6GALNAC3-For: TGCCATTTCTTCTCCATTCC
ST6GALNAC3-Rev: CCAAGCAAAAAGGAAAGCTg
PRODUCT SIZE: 5972,

TBC1D3H-For: gcgccccatgtttattacaa
TBC1D3H-Rev: AGCTGCTATCCCCTACAGCA
PRODUCT SIZE: 3346,

ROBO2-For: atgcttccagcagccttaaa
ROBO2-Rev: AGCAAGGCATTTTCCAGCTA
PRODUCT SIZE: 5600,

KCNIP4-For: AGCTGAGGCAAGACCGAATA
KCNIP4-Rev: cccttgctgccttacagtct
PRODUCT SIZE: 4208

PACRG-For: aaacagggcagtcatggaac
PACRG-Rev: ctgtgcccagccagataaat
PRODUCT SIZE: 5214

CSMD1-For: AACTGACCGGCCACTTTCTA
CSMD1-Rev: TGAGCCCTGGAATCTTGTCT
PRODUCT SIZE: 1559,

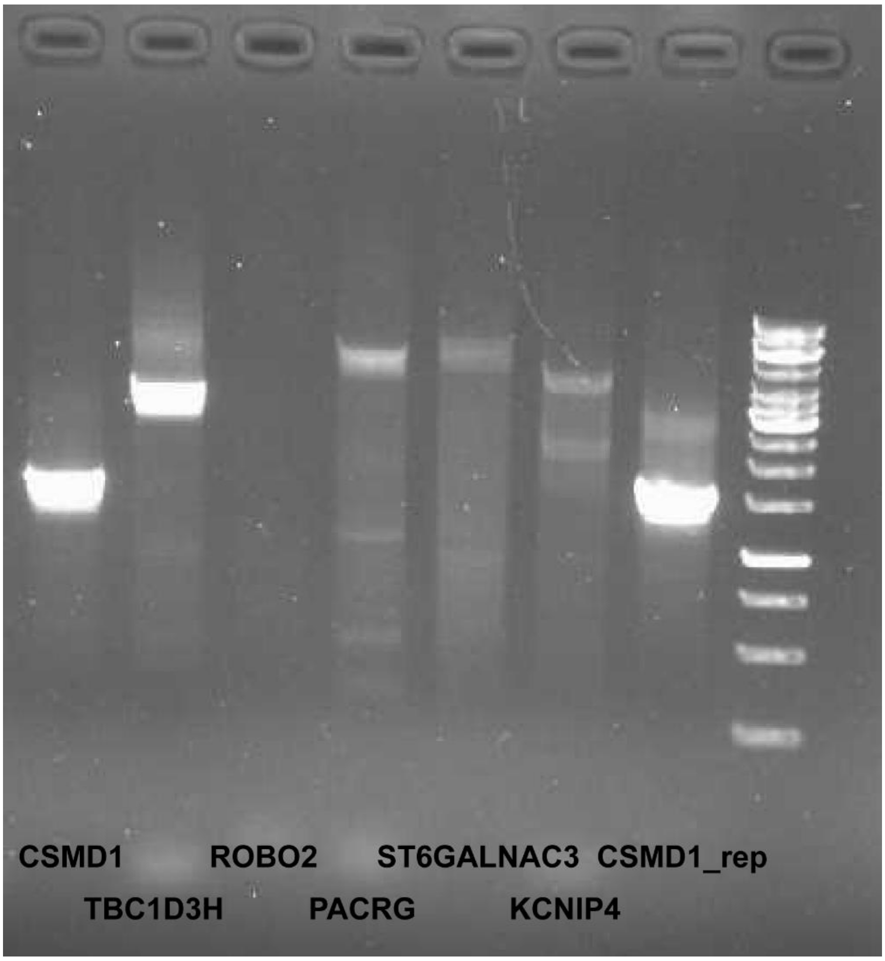

Supplementary Figure 1. The PCR amplified gDNAs of top unstable methylation genes and tumor suppressor genes. Gel indicating PCR fragments amplified from various genes by use of the general primer pairs.

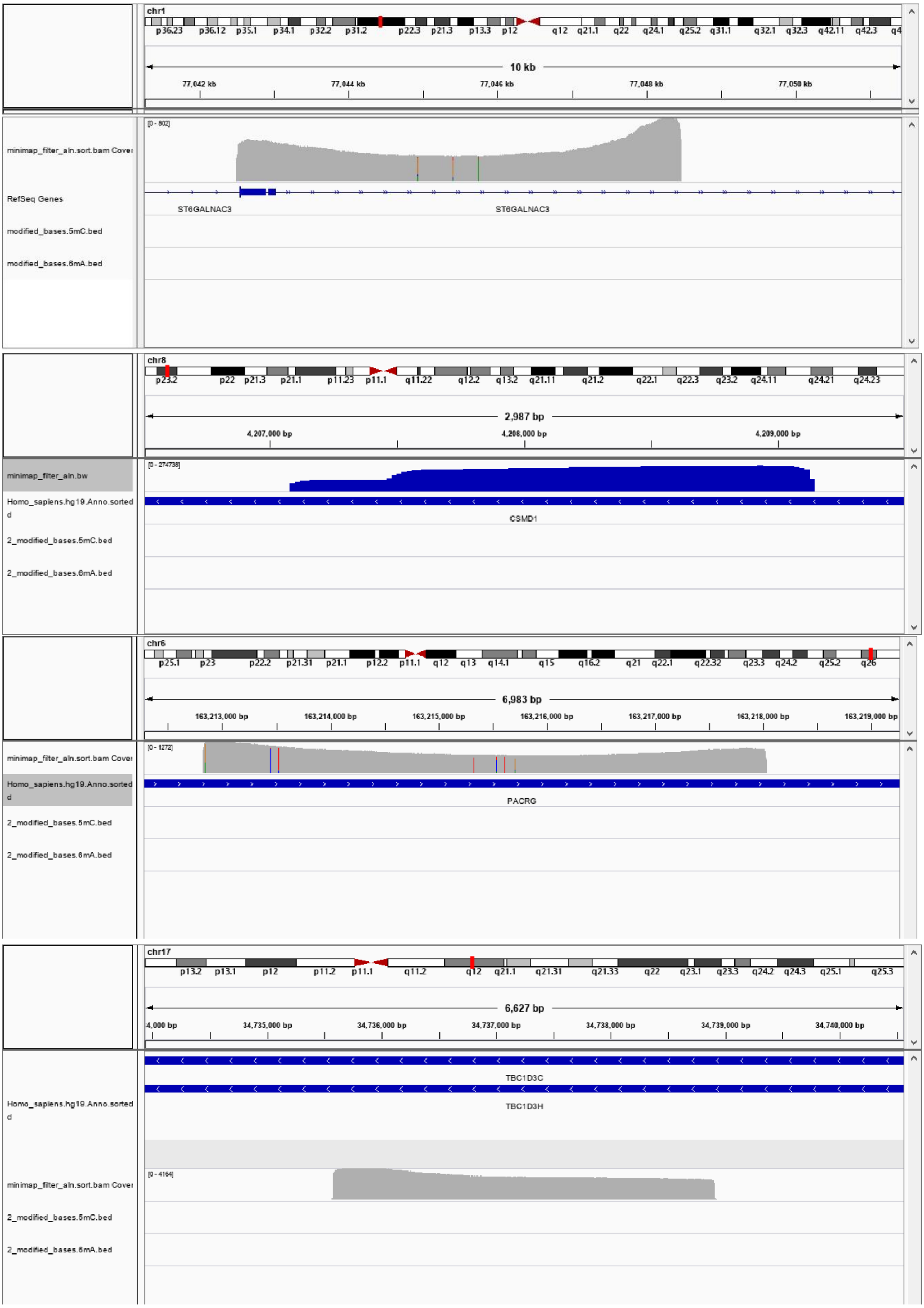

Supplementary Figure2. The output of the PCR amplified gDNAs of top unstable methylation genes. After the Nanopore sequencing, we performed the same analysis. The sequence after PCR amplified should not have methylation sites. It proved the reliability of our data processes.
